# Spatial Niche Facilitates Clonal Reproduction in Seed Plants under Temporal Disturbance

**DOI:** 10.1371/journal.pone.0116111

**Published:** 2014-12-30

**Authors:** Shin Fukui, Kiwako S. Araki

**Affiliations:** 1 Center for Ecological Research, Kyoto University, Otsu, Shiga, Japan; 2 Agro-Meteorology Division, National Institute for Agro-Environmental Sciences, Tsukuba, Ibaraki, Japan; 3 Department of Biotechnology, Faculty of Life Sciences, Ritsumeikan University, Kusatsu, Shiga, Japan; University of A Coruña, Spain

## Abstract

The evolutionary origins and advantages of clonal reproduction relative to sexual reproduction have been discussed for several taxonomic groups. In particular, organisms with a sessile lifestyle are often exposed to spatial and temporal environmental fluctuations. Thus, clonal propagation may be advantageous in such fluctuating environments, for sessile species that can reproduce both sexually and clonally. Here we introduce the concept of niche to a lattice space that changes spatially and temporally, by incorporating the compatibility between the characteristics of a sessile clonal plant with its habitat into a spatially explicit individual-based model. We evaluate the impact of spatially and temporally heterogeneous environments on the evolution of reproductive strategies: the optimal balance between seed and clonal reproduction of a clonal plant. The spatial niche case with local habitats led to avoidance of specialization in reproductive strategy, whereas stable environments or intensive environmental change tended to result in specialization in either clonal or seed reproduction under neutral conditions. Furthermore, an increase in spatial niches made clonal reproduction advantageous, as a consequence of competition among several genets under disturbed conditions, because a ramet reached a favorable habitat through a rare long-distance dispersal event via seed production. Thus, the existence of spatial niches could explain the advantages of clonal propagation.

## Introduction

Clonal reproduction is a universal mode of reproduction used by a broad range of terrestrial organisms [1]–[3]. This reproductive mode is described as the asexual way of propagating, and is often compared with sexual reproduction. Both sexual and asexual modes of reproduction have their respective benefits: the former produces genetically diverse individuals via genomic recombination, while the latter produces offspring without the need for a mating partner [2], [4]. The evolution and maintenance of sexuality has long been the subject of debate about its relative costs and benefits [5], [6]. Several hypotheses have been proposed, such as Muller’s ratchet [7] and the deterministic mutation hypothesis [8], which suggest that sexuality can remove harmful genes, and the Red Queen hypothesis [9], which suggests that sexuality enables species to escape from infectious diseases by virtue of their genetic diversity. Despite the importance of the question, there have been few studies testing these hypotheses that use experimental approaches [10]–[12], so these hypotheses are still competing with one another.

Many taxonomic groups include species that reproduce both sexually and asexually, and their modes of propagation are tightly connected to the dispersal of their offspring. For example, several seed plants (spermatophytes) produce not only seeds but also clonal offspring from vegetative organs. New colonies of corals such as *Plexaura kuna* and *Montastraea annularis* are founded either clonally by fragments of colonies, or by offspring from egg spawning (inseminated gametes) [13], [14]. In the case of ant species such as *Wasmannia auropunctata*, *Vollenhovia emeryi*, and *Paratrechina longicornis*, colonies expand to neighboring areas by means of asexually produced queens and nest budding, while workers are sexually produced and are therefore genetically diverse [15]–[17]. Asexually produced clonal offspring generally disperse to closer places than sexually produced ones. Despite the absence of genetic variation and the limited migration distance, clonal reproduction has continued successfully in combination with sexual reproduction in many species of sessile organisms.

Here we focus on clonal reproduction in seed plants. Clonality has evolved independently several times and has remained a dominant trait in various phylogenetic lineages [18], [19]. Actually, 70–80% of herbaceous plants in the temperate zone have multiple reproductive modes [18], [20]. On account of their rooted lifestyles, clonal offspring grow around their parent plants [4], [21], [22]. Consequently, genetically identical but phenotypically independent individuals (called ramets) of various ages are clustered and live together (this unit is called a genet) in the same space for a long time in a population. It is therefore natural that they experience not only various environmental changes and/or attacks by herbivores and pathogens [23]–[25] but also demographic changes of the species during their lives [26], [27]. Sexual reproduction works well against unpredictable environmental fluctuation by providing long dispersal distances and genetic diversity [28], [29]. It is thus still an unanswered question why clonal plants have evolved and what mechanisms work to maintain clonal reproduction under such conditions.

Competition among generations can be understood as an issue affecting the evolution of dispersal strategies: seed reproduction is the long-distance dispersal strategy, and clonal reproduction is the short-distance one. Hamilton and May [30] demonstrated that the long-distance dispersal of newborn offspring at a certain rate was an evolutionarily stable strategy (ESS) even if there was no competition between a parent and its offspring. Furthermore, a small disturbance of habitat makes short-distance dispersal advantageous, whereas a large disturbance makes long-distance dispersal advantageous, if resource allocation to each dispersal strategy is fixed [31]. Nakamaru et al. [32] demonstrated, using the colony-based lattice model, that a disturbance affecting a large area of habitat and occurring at high frequency favored a long-distance dispersal strategy, whereas a disturbance causing damage within a small area at low frequency made short-distance dispersal more advantageous. As regarding dispersal of offspring, their model framework is applicable to seed plants because the mode of offspring dispersal is similar to that in ant colonies; the long- and short-distance dispersal strategies correspond to clonal and sexual reproduction of plants [31], [33], and colony size correlates with plant size. On the other hand, the impact of spatial heterogeneity of habitat on dispersal strategy is completely different, because seeds of plants do not choose the place where they germinate, but land there by chance, unlike animals, who can choose their habitat by moving. In fact, while animals can move to favorable habitats, the movement of sessile organisms is restricted within a limited distance and depends on other mediators. Thus, both spatially and temporally, environmental heterogeneity should be important keys to the evolutionary processes behind the development of the reproductive strategies of seed plants.

To investigate the direction of selective pressures on the reproductive strategy of sessile organisms, we have developed a lattice model that takes into account the spatial niche effect and temporal disturbance. In particular, we examine whether clonal reproduction is as effective as that via seeds in seed plants, without considering physiological integration and division of labor. Clonal reproduction should be a reasonable strategy if the habitat is constant, because genets would be spared the cost of unifying the connected organs. On the other hand, clonal reproduction causes intra-genet competition if each ramet interferes with the other ramets for resources [26], [33], which also influences inter-genet competition [34], [35]. We define “spatial niche” as spatial habitat heterogeneity, and environmental change of a habitat as equivalent to temporal heterogeneity. Then, because we suppose phenotype is genetically fixed in each individual, an individual plant with the optimal genotype colonizes a certain niche (thus, the “neutral case” as the case with no niche concept of habitat heterogeneity and plant phenotype). We evaluate the effect of spatial niche itself on the evolution of reproductive strategies by including and excluding this effect and comparing the results.

## Methods

### Simulation Framework

**Figure 1:**
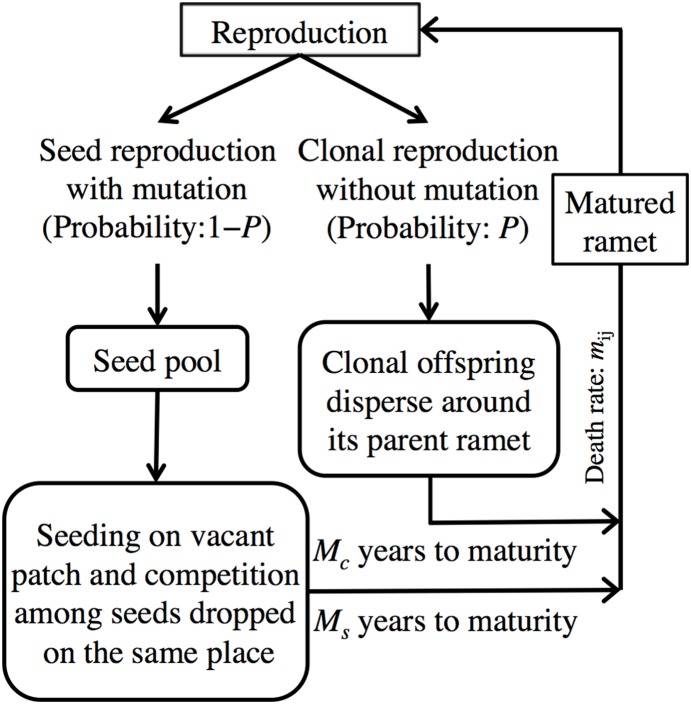
The flow chart for the spatially explicit individual-based simulation of plant dynamics.

The model is a spatially explicit individual-based (SEIB) model in which each individual grows on a lattice space arranged in a torus form. Each lattice site is empty or occupied by a single plant. We model growth, reproduction, dispersal, competition, and death as life history events, and disturbance as a stochastic one. The plant species in our model are assumed to be perennials that perform clonal and seed reproduction, and all events occur in an annual step, as illustrated in Fig. 1. Several life history traits and environmental characters are described by model parameters, which are summarized in Table 1. Plants can propagate after they reach the age of maturity, and they produce offspring by clonal reproduction with probability *P,* or via seeds with probability (1 – *P*). If a plant chooses clonal reproduction, an offspring can occupy one of the surrounding eight neighboring lattice sites around its parent (Moore neighborhood), with probability *P*/8, contingent upon the cell being empty. If a plant chooses seed reproduction, a parent plant produces *N* seeds, and all seeds from all plants in the lattice collect in the same seed pool. Occupation of a vacant patch by a clonal offspring, next to its parent ramet, occurs first, after which the residual empty sites are available to seeds, which can reach every vacant site. It takes *M_c_* years and *M_s_* years for the clonal offspring and seeds to mature. In accordance with the hypothesis that abundant resource translocation is an important advantage for clonal offspring in the initial growth stage, we assume that clonal offspring reach maturity faster than seed offspring (*M_s_*>*M_c_*). We assume the number of seeds (*N*) to be constant per individual and, for simplicity, ignore the gradient in seed density related to dispersal distance from the parent. Several seeds can settle into the same lattice site, and then the competition among them selects the fittest one (the way in which competition operates will be described later).

**Table 1:** Parameters in the model.

Parameters	Description
***P*** **_ij_**	The reproductive strategy of a plant at site (i, j).(100% clonal reproduction if *P* = 1, and 100% seed reproduction if *P* = 0.)
***Q*** **_ij_**	The trait suitable for the habitat of a plant at site (i, j).
***µ***	The mutation rate for the genotypes *P* and *Q*.
***M*** **_c_**	Years to maturity for clonal offspring.
***M*** **_s_**	Years to maturity for offspring grown from seed.
***d*** **_min_**	The basal death rate of a plant.
***m*** **_ij_**	The death rate of a plant at site (i, j) including the effect of compatibility between habitat and plant traits.
***N***	The number of seeds produced by a parent at every opportunity for seed production.
***E*** **_ij,_**_***t***_	The habitat characteristics at site (i, j) at time *t.*

### Environmental heterogeneity as spatial niche

**Figure 2:**
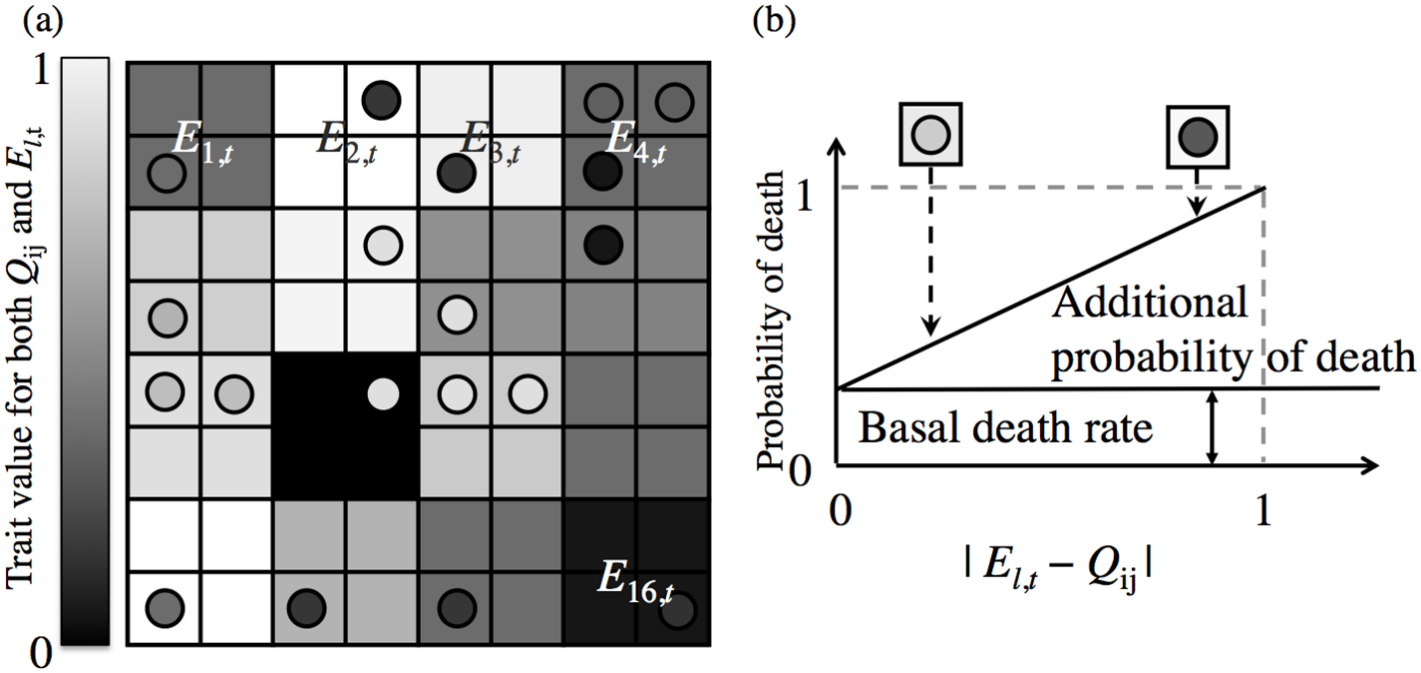
The visual concept of spatial heterogeneity on the habitat lattice and the plant mortality rate . The figure (a) represents the concept of spatial heterogeneity. The grey scale in the squares represents the trait value (0–1) of the habitat: the value for the pure white habitat is zero and that for the pure black habitat is one. In this case, there are 16 different habitats (*E*_1,*t*_, *E*_2,*t*_,…*E*_16,*t*_) within the total lattice space and each habitat has 2×2 square sites. The grey scale in circles represents the plant trait value (*Q*_ij_). The similarity of the grey scale between *E*_l,t_ and *Q*_ij_ determines the death rate of the individual plant inhabiting (i, j), and its relationship is illustrated in (b). The two combinations of square and circle are the example of the difference between habitat and plant trait values in (b).



The predicted death rate in our model consists of two components: one is the basal death rate determined for the species and the other is the additive probability of death depending on the compatibility with the growth environment (niche). Here we generate several habitat environments by dividing the lattice space into *k* areas. As shown in Fig. 2 (a), each area is assigned environmental conditions associated with a particular habitat. The boundary of each habitat is contiguous its neighboring habitats, so that clonal offspring of a mature plant inhabiting the edge of a certain habitat can colonize the edge of another adjacent habitat. The disturbance in our model changes aspects of the habitat environment such as soil moisture and/or light intensity. This is represented by the value of the environmental condition of that habitat changing from *E_t_* to *E_t+_*_1_ with an associated probability of *p*. If a habitat is disturbed at time *t*, the value *E_t_*_+1_ is taken from the Gaussian distribution with mean *E_t_* and variance *q*. This is described mathematically as:

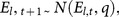
(1)where *l* represents a certain habitat (1≤*l*≤*k*) and the value of *E* lies between zero and one. Change in the value of a habitat affects the plant death date indirectly via change in habitat condition, so that the magnitude of environmental change *q* corresponds to the plant death date.

The environmental condition of a site (*E*) and the genotype of an individual (*Q*) inhabiting that site determine the death probability of the individual as shown in Fig. 2 (b). Both variables are continuous numerical values between zero and one, and the difference between a plant and its habitat results in an additive probability of death as follows:
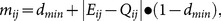
(2)where i and j represent the position on the lattice, *m*_ij_ is the death probabiligy of a plant living at site (i, j), and *d*_min_ represents the basic death rate. Each plant survives every year with probability 1 – *m*_ij_. The environmental changes indirectly affect the plant death rate. We conduct the simulation under several changes in environmental conditions, altering the frequency (*p*) and the magnitude (*q*) of environmental change, with several levels of environmental heterogeneity (number of different habitats, *k*).

### Mutation of plant traits and reproductive strategy

A plant has two heritable traits: one is the reproductive strategy *P* and the other is the trait of suitability for the habitat *Q*. Each of these traits is represented as a numerical value between zero and one. Mutation of the traits is expressed as changes in these values in seeds. Here genetic recombination via sexual reproduction is simply expressed as mutation in order to focus on the difference from clonal reproduction, which produces no genetic variation. In the same way as for *E*, the genetic traits of the next generation produced via seed reproduction are taken from the Gaussian distribution as follows:
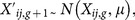
(3)where *g*, *X*_ij_, *X*′_ij_, and *µ* represent the traits of the parent generation (*X* ∈ *P, Q*), those of the next generation, and the mutation rate, respectively. Each trait undergoes mutation independently. Depending on the difference between *E*_i’j’_ and *Q*′_i’j’_, the best-fit seed for a habitat can be determined if several seeds drop into the same site (i’, j’). When this occurs, the offspring with the lowest death probability will survive.

### Neutral environment as control

We also model the case in which the habitat has no spatial heterogeneity. Our object in this study is to reveal the impact of considering the effect of the spatial niche on the evolution of clonal reproduction. Comparing the spatial niche and neutral cases highlights the effect of inter-genet competition, because in the neutral case the habitat compatibility phenotype is meaningless, i.e. inter-genet competition can be ignored. The neutral situation, in which all genotypes have an equal ability to grow, reproduce and survive, is important, together with the number of niches, for evaluating the effect of habitat heterogeneity. Thus, we remove the additional probability of death caused by the difference between habitat and plant traits. Therefore, the baseline death rate is held as *d*_min_ for all plants. On the other hand, the effect of environmental change on the reproductive strategy should still be acting. An increase in death rate caused by environmental change occurs according to the age of each plant. A recent arrival suffers no increase in probability of death, but an old individual in the same habitat does have an increased probability of death, derived from the change in compatibility between the habitat and the plant’s traits following environmental change.

### Simulation Settings

We ran the simulation under several spatially and temporally heterogeneous conditions. The variables used in the simulations are described in Table 2. We generated different environments by dividing the total lattice space (100×100 square sites) into (1) 25 habitats (20×20 square sites), (2) 16 habitats (25×25 square sites), and (3) 4 habitats (50×50 square sites). The temporal heterogeneity was caused by environmental changes, which occurred at different frequencies (*p*) and with different magnitudes (*q*) as follows: (*p*, *q*) = (0, 0), (0.01, 0.01), (0.01, 0.1), (0.1, 0.01), and (0.1, 0.1). For basic plant life history, we set the time to mature for a seedling as five years (*M_s_* = 5), and as three years for clonal offspring (*M_c_ = *3), and the basic death rate as 0.2 every year (*d*_min_ = 0.2). The number of seeds produced by a parent was set as 100 (*N = *100)**.** The initial plant traits were randomly chosen from a uniform distribution independently of habitat condition, and the initial population covered 90% of total sites on the lattice. One hundred simulations were conducted and each simulation was run for 10,000 years. After running the simulation, the reproductive strategy (*P*) was collected from the remaining plants, and then its frequency distribution was calculated. The mutation rate *µ* was fixed at 0.01 throughout this study.

**Table 2:** Variables in the model.

Variables	Description
***p***	The frequency of environmental change.
***q***	The magnitude of environmental change.
***k***	The number of different habitats (environmental heterogeneity) within the total lattice space.

In this simulation setting, the habitat space was finite, so an increase in the number of habitats, i.e. an increase in environmental heterogeneity within the total lattice space, resulted in a decrease in the space occupied by each habitat. In contrast, the number of potential seed reproduction events increased as the heterogeneity increased. Thus, we also examined the case in which the habitat size was fixed at 20×20 square sites but the heterogeneity (*k*) differed, which meant that the total lattice space became larger as the habitat heterogeneity increased. Concretely, the total lattice size is 40×40 square sites when *k* = 4, and it is 100×100 square sites when *k* = 25.

## Results

### Effects of temporal heterogeneity of environment

**Figure 3:**
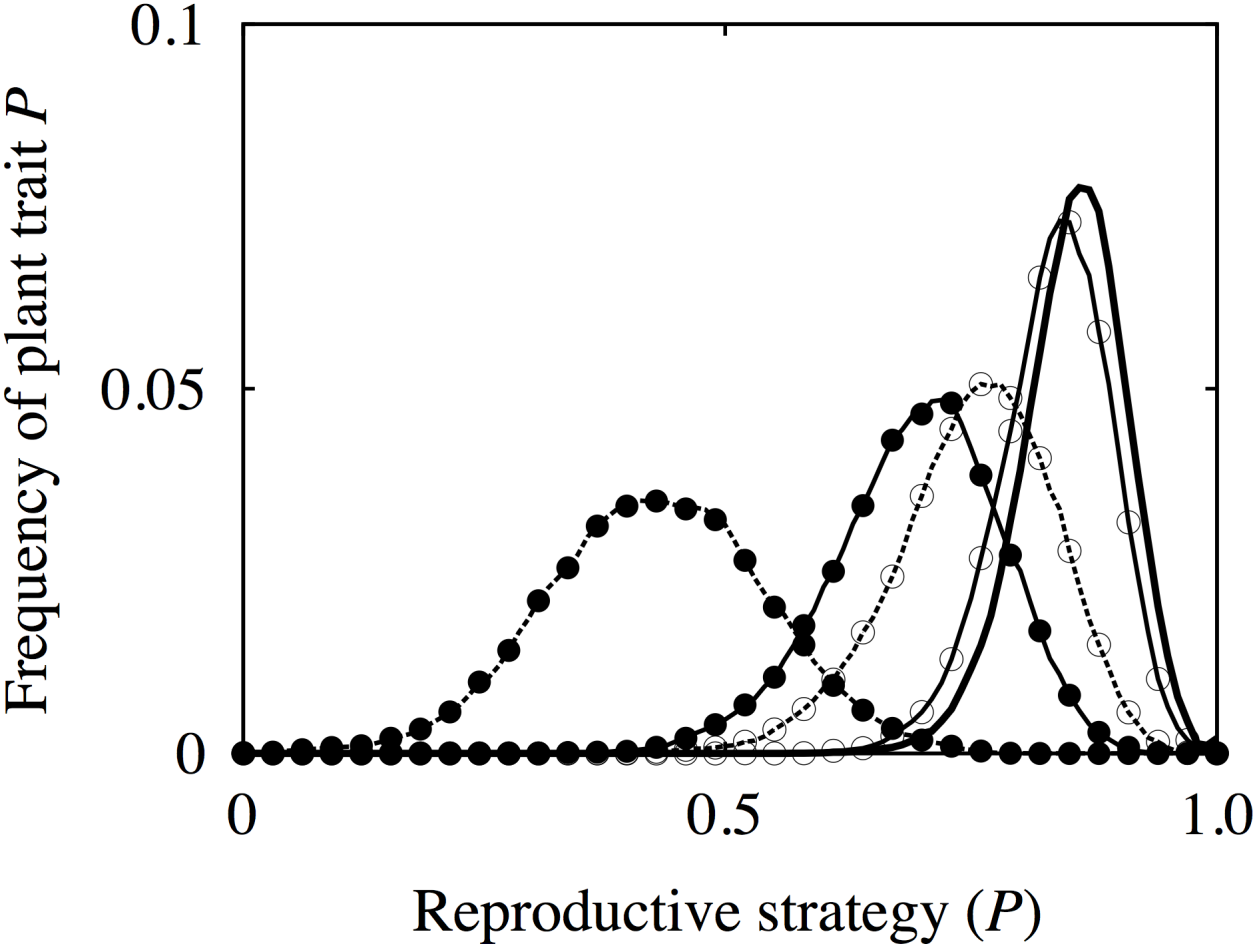
Several patterns of the frequency distribution for reproductive strategies. The horizontal axis represents the reproductive strategy (*P*) of an individual plant (0: seed reproduction only, 1: clonal reproduction only), and the vertical axis represents the frequency of each value of *P* in the plant population. This depends on the degree of environmental change in a habitat and the number of spatial niches. The number of habitats is fixed at *k* = 16. The values describing the environmental change for each line are: thick solid line corresponds to (*p*, *q*) = (0, 0), solid line with open circles corresponds to (*p*, *q*) = (0.01, 0.01), solid line with close circles corresponds to (*p*, *q*) = (0.01, 0.1), dotted line with open circles corresponds to (*p*, *q*) = (0.1, 0.01), and dashed line with close circles corresponds to (*p*, *q*) = (0.1, 0.1).

First, we demonstrated how the temporal heterogeneity affected the evolution of reproductive strategy, by varying the frequency and magnitude of environmental change under the condition that spatial heterogeneity was fixed at *k* = 16. Fig. 3 shows the change in frequency distribution of the reproductive strategy (*P*), depending on the values of *p* and *q*. As suggested in previous studies, environmental change favored seed reproduction. In contrast, clonal reproduction became advantageous if the habitat environment was stable but empty spaces remained available for long-distance dispersal, as Hamilton and May [30] indicated. The width of the frequency distribution differed depending on the environmental condition of a habitat, and it increased as environmental change occurred more intensively. The balance between the advantage gained by rapid spread by seed dispersal into new habitats following environmental change and the advantage of strong clonal propagation with a suitable trait for its growth habitat determined the shape of the distribution. In an intensively disturbed environment, both modes of reproduction had beneficial effects for the spread of population. We also checked the effect of changing *N* (number of seeds) and *M*_c_ (age of maturity for clonal offspring). An increase in *N* shifted the frequency distribution towards seed reproduction on the whole, while an increase in *M*_c_ shifted it towards clonal reproduction on the whole.

### Effect of spatial niche compared with the neutral condition

**Figure 4:**
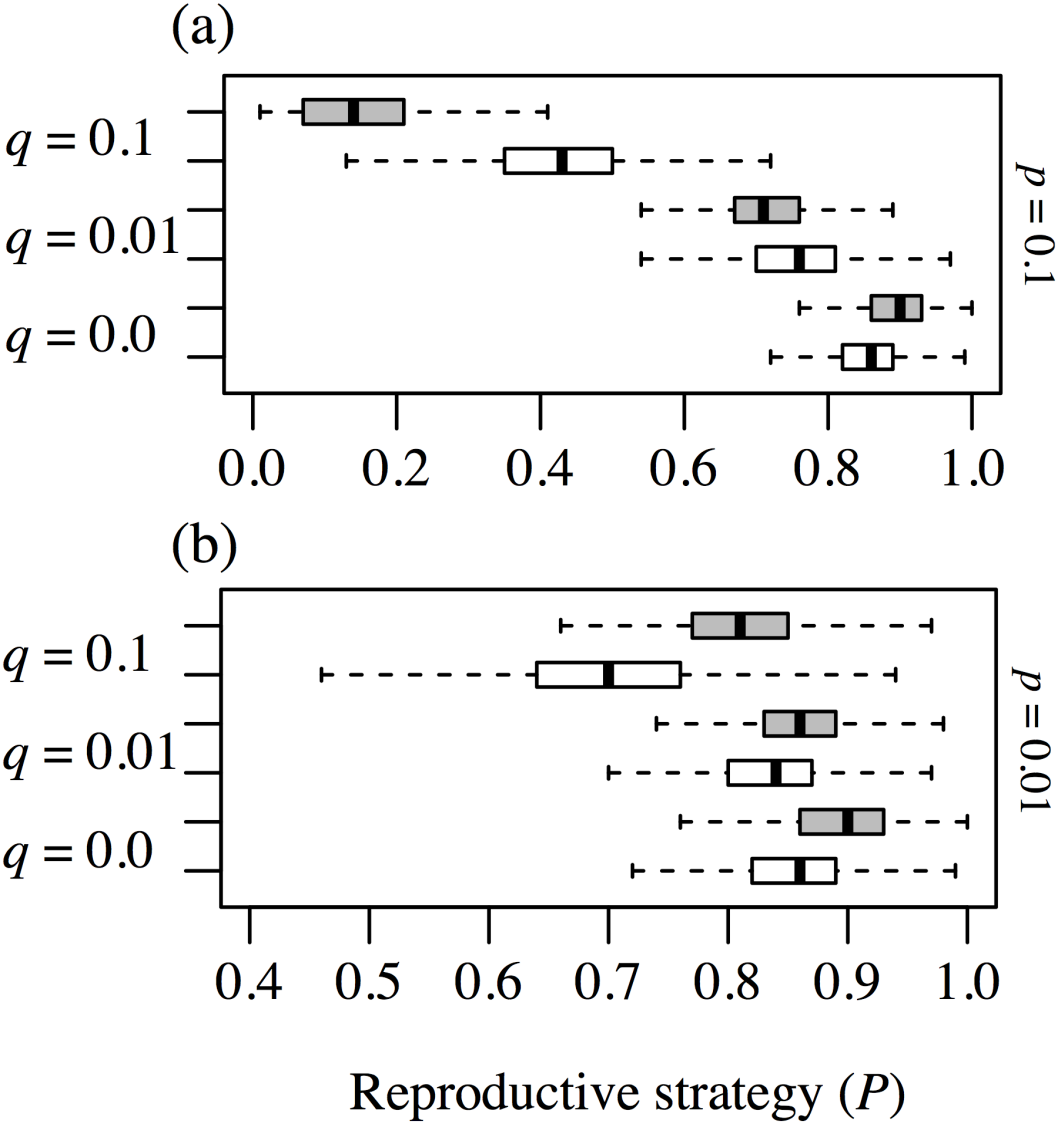
The changes in reproductive strategies in response to environmental change, comparing the spatial niche and neutral cases. The spatial heterogeneity is fixed at *k = *16, and the white boxplot represent the spatial niche case and the grey boxplots represent the neutral case. Panel (a) represents the case in which frequency of environmental change is high, i.e., *p = *0.1, and panel (b) represents the case of low frequency of environmental change (*p = *0.01). Within each panel, the difference of the magnitude of environmental change is illustrated: *q = *0.1 at the top, *q = *0.01 in the middle, and *q* = 0 at the bottom. When *q = *0, it is identical to *p = *0, because it means there is no environmental change.

Fig. 4 shows how the reproductive strategies responded to environmental change of a habitat with the same settings as in Fig. 3 (*k* = 16) in the spatial niche case (white) and the neutral case (grey). When the frequency of environmental change was low (*p* = 0.01, Fig. 4 (b)), reproductive strategies shifted from clonal reproduction toward seed reproduction as the magnitude of the change increased in both cases. The neutral environment favored clonal reproduction more than the environment with habitat heterogeneity, under all environmental change conditions. When the frequency of environmental change was high (*p* = 0.1, Fig. 4 (a)), the strategy became extreme: clonal reproduction became more advantageous in the absence of environmental change (*q* = 0.0) whereas seed reproduction was more favorable with intense change (*q* = 0.1). The reason for this is that environmental heterogeneity worked as a barrier that restricted a genet moving into its suitable habitat. A high frequency of environmental change caused many empty sites throughout the lattice, so that long-distance dispersers were more successful than short-distance dispersers in both cases. On the other hand, a high magnitude of disturbance killed many individuals at the same time and provided a good opportunity to a clonal offspring if its parent had survived, so that clonal reproduction had a greater effect in spreading the population after environmental change.

### Effect of spatial heterogeneity of environment

**Figure 5:**
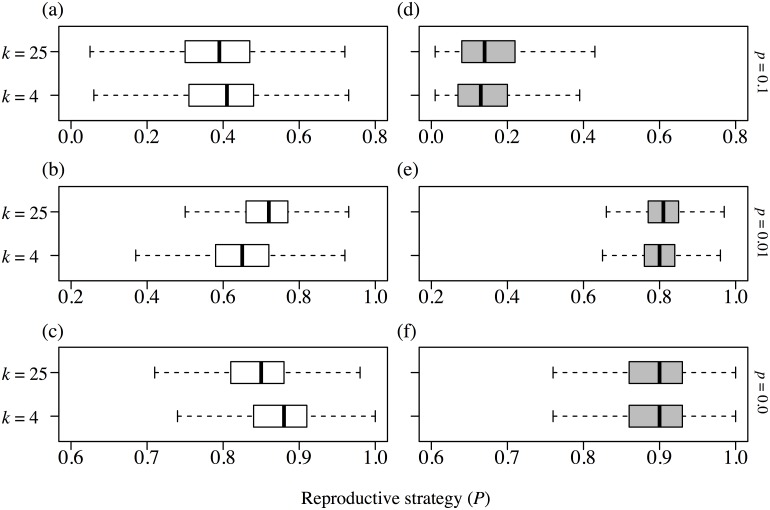
The frequency distributions of reproductive strategies in both the spatial niche and the neutral cases. The frequency of environmental change is fixed at *q = *0.1. The left side panels (a–c) represent the spatial niche case and the right side panels (d–f) represent the neutral case. The three layers of panels represent the different magnitudes of change: *p = *0.1 in the top panels (a, d), *p = *0.01 in the middle panels (b, e), and *p = *0 in the bottom panels (c, f). Within each panel, habitat heterogeneity is indicated: *k = *25 for the upper boxplot and *k = *4 for the lower boxplot. White boxplots represent the spatial niche case and the grey boxplots represent the neutral case.

We demonstrated the effect of spatial heterogeneity (*k*) on reproductive strategy (Fig. 5). Here we show the case in which the frequency of environmental change was large (*q = *0.1) because in this case the effect of environmental change was clear, as shown in Fig. 4. Habitat heterogeneity made seed production advantageous in the absence of environmental change (*p = *0.0, *q = *0.0, Fig. 5 (c)), and environmental change made seed production advantageous regardless of habitat heterogeneity, but in the spatial niche case the impact was different depending on the degree of environmental change. Increasing habitat heterogeneity tended to make clonal reproduction more advantageous with intermediate environmental change (*p = *0.01, *q = *0.1, Fig. 5 (b)), whereas intensive environmental change made the difference in heterogeneity unclear (*p = *0.1, *q = *0.1, Fig. 5 (a)). In contrast to the spatial niche case, there was no great distinction among habitat heterogeneities in the neutral case (right column in Fig. 5).

**Figure 6:**
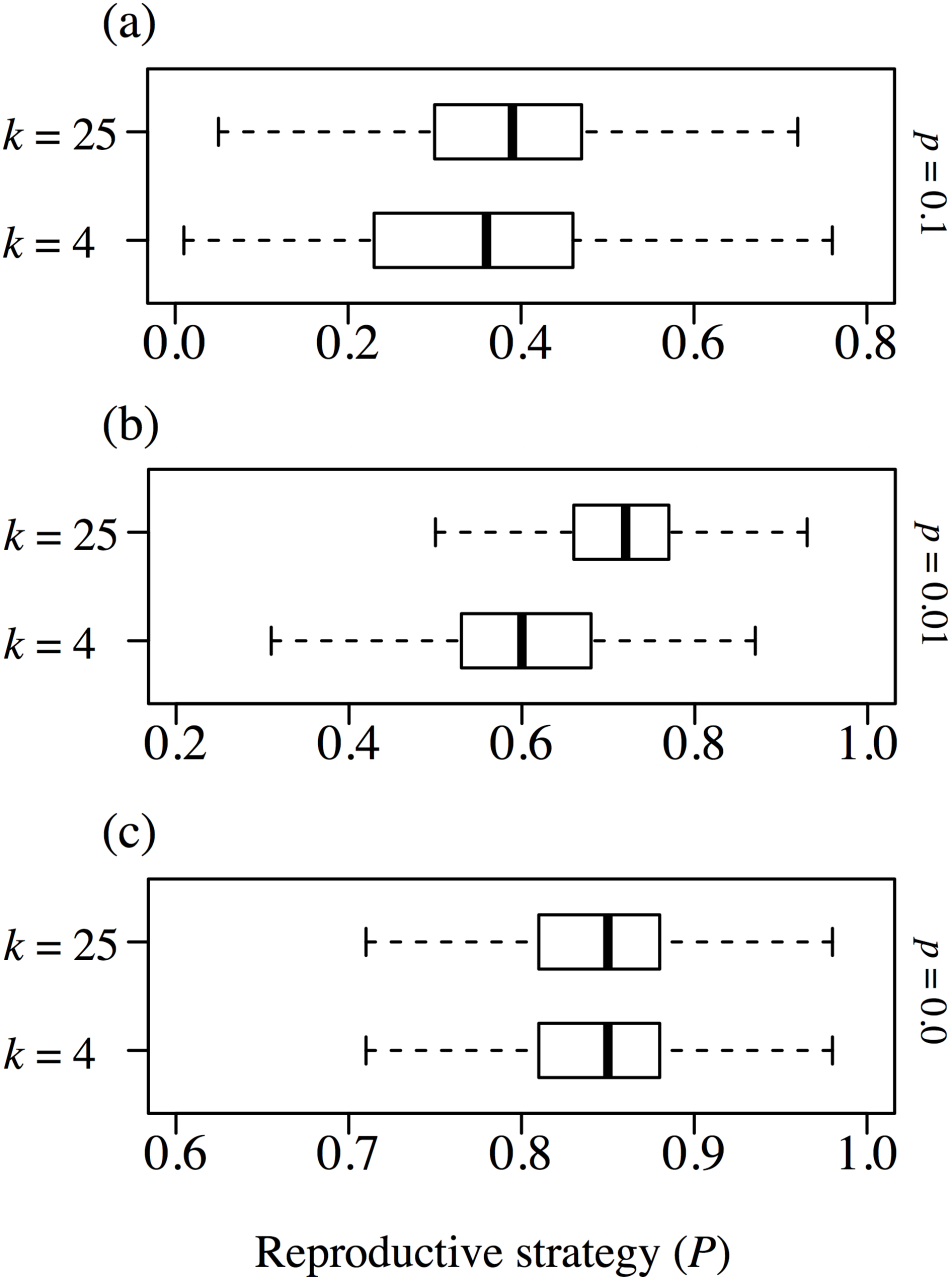
The frequency distributions of reproductive strategies under several levels of spatial heterogeneity, with fixed habitat sizes. The frequency of environmental change is fixed at *q = *0.1. The three panels represent the different magnitudes of disturbance: *p = *0.1 in (a), *p = *0.01 in (b), and *p = *0 in (c), as in Fig. 5. Within each panel, habitat heterogeneity is indicated: *k = *25 for the upper boxplot and *k = *4 for the lower boxplot.

Next, we demonstrated the effect of spatial heterogeneity (*k*) on reproductive strategy, excluding the effect of difference in size of the lattice within a habitat. The effect of intra-genet competition was identical among habitats, but the opportunity for seed colonization was lower than when total lattice space was fixed. There was no clear difference in the reproductive strategy under a stable environment (Fig. 6 (c)), but lower habitat diversity favored seed reproduction in the case of intermediate environmental change (Fig. 6 (b)). Also, there was no clear difference in reproductive strategies among habitat conditions with intensive environmental change (Fig. 6 (a)) or in the case that the total lattice size was fixed (Fig. 5 (a)). Environmental heterogeneity in the neutral case did not cause any difference in the reproductive strategy (results not shown) or in the case that total habitat was fixed (Fig. 5 (a−c)). It was quite natural that clonal propagation should be unfavorable when the size of each habitat was small. The number of empty sites should increase if the total habitat area is enlarged, so the possibility for seeds to settle into vacant sites will also increase. It should be noted that an increase in habitat heterogeneity drove the reproductive strategy toward seed reproduction in both the case of fixed habitat size and the case of fixed total lattice size.

## Discussion

### Comparison between spatial niche and neutral models

This study demonstrates that the presence of spatial niches alters the impact of environmental change on the habitat condition, relative to the neutral case (Fig. 4). It shows that the effect of environmental change on reproductive strategy is almost the same in both the spatial niche and the neutral cases, meaning that long-distance seed dispersal is effective under a highly changed environment (Fig. 3), as previous studies have concluded [31], [36], [37]. In the spatial niche case, however, a high frequency of environmental change makes clonal reproduction more advantageous than in the neutral case. In other words, compatibility with the habitat makes selective pressure favor clonal reproduction (Fig. 4). Furthermore, regarding the direction of selective pressure, the long-distance dispersal strategy is more advantageous under low frequency and large environmental change in the spatially heterogeneous condition than in the neutral condition (Fig. 4). This implies that inter-genet competition tends to favor the long-distance dispersal strategy under spatial heterogeneity.

Natural habitats are never homogeneous spatially or temporally [38], [39], although results of previous studies are consistent with the neutral case results of this study. On the forest floor, for example, light strength and soil moisture change with time due to regeneration of trees [40], [41]. Advantages of clonal reproduction under conditions of disturbance are discovered by considering habitat heterogeneity and the adjustment between it and the phenotype of each individual. As this result suggests, habitat heterogeneity as it relates to the fitness of an individual has a great impact on the life history strategy and/or biodiversity. However, there are almost no studies that incorporate the spatial niche effect, except that of Tubay et al. [42], who investigated the biodiversity of phytoplankton in an aquatic ecosystem. It would therefore be useful to investigate biodiversity in an ecosystem with spatial heterogeneity, as opposed to in uniform (neutral) ecosystems [43].

### Effects of spatial and temporal variation on reproductive strategy

The results obtained here under various spatial niche environments (Figs. 5, 6) reveal the evolutionary effects of intra-genet competition. When no environmental change occurs, seed (i.e., sexual) reproduction becomes more advantageous, because it avoids intra-genet competition within the same habitat (Fig. 5). This also indicates that genetic diversity maintained by sexual reproduction can deal with variable habitats [44], [45], especially when seeds escape outward from already-filled niches in this model. On the other hand, when environmental change occurs in a spatially heterogeneous habitat, seed reproduction is less advantageous and clonal reproduction becomes beneficial (Figs. 5 (b) and 6). Spatial heterogeneity provides similar environments within the total habitat, and consequently the clonal reproducer can spread its population into new areas when rare opportunities for long-distance dispersal occur. This pattern is inconsistent both with the theory that genetic diversity gives seed reproduction an advantage and with previous studies showing that seed reproduction is advantageous under changed environments [24], [46]. However, several clonal plants have adapted and been favored at the early successional stage [47], with dynamics similar to those found in our simulation. Generally, maintaining genetic diversity via seed reproduction tends to become an effective strategy in a fluctuating environment, such as one subject to disturbance [31], [32], [36], [37]. However, once an individual is rooted in a suitable patch, it can spread circumferentially by vegetative propagation under a relatively stable environment [33], because environmental conditions are generally relatively similar in neighboring habitats. In other words, if habitat conditions are suitable, clonal reproduction is more effective because of the rapid propagation that is possible during the early stages of the young plants’ lives. Actually, several pioneer or invasive plant species (for example, *Miscanthus sinensis* and *Fallopia japonica*) that rapidly colonize open spaces have clonal-propagating abilities, which indicates an adaptive response to good patches that appear after environmental change occurs. In branching scleractinian and gorgonian corals exposed to a wave-disturbed environment, new colonies are founded predominantly by fragments of broken colony branches, not by inseminated gametes that can emigrate long distances [13], [14].

Since the probability of seed establishment decreases according to the distance of dispersal from the parents, density effects on the same (homogeneous, similar) habitat become larger in practice [48], [49]. Furthermore, the situation in which it is difficult for clonal offspring to migrate to different patches because of the difference in environment is similar to the situation of habitat fragmentation. Travis and Dytham [50] considered the effect of habitat fragmentation on dispersal strategies, and showed that long-distance dispersal was more advantageous as habitat size became smaller in the SEIB model. Heibeler [51] examined unfavorable places to live on the lattice space, and demonstrated that habitat fragmentation favored long-distance dispersal, whereas a clustered habitat favored short-distance dispersal. Long-distance dispersal can be advantageous within a clustered habitat in some cases, but the opposite has never been demonstrated. Nevertheless, clonality would become advantageous as long as there is diversity in the habitat environments that an individual reaches.
